# Antibiofilm Activity of *Azadirachta indica* and *Catharanthus roseus* and Their Synergistic Effects in Combination with Antimicrobial Agents against Fluconazole-Resistant *Candida albicans* Strains and MRSA

**DOI:** 10.1155/2022/9373524

**Published:** 2022-03-21

**Authors:** David Neglo, Francisca Adzaho, Irene A. Agbo, Richmond Arthur, Daniel Sedohia, Clement Okraku Tettey, Sayanika Devi Waikhom

**Affiliations:** ^1^Department of Basic Sciences, School of Basic and Biomedical Sciences, University of Health and Allied Sciences, PMB 31, Ho, Ghana; ^2^Department of Biomedical Sciences, School of Basic and Biomedical Sciences, University of Health and Allied Sciences, PMB 31, Ho, Ghana; ^3^Institute of Traditional and Alternative Medicine, University of Health and Allied Sciences, PMB 31, Ho, Ghana; ^4^Department of Clinical Microbiology, Kwame Nkrumah University of Science and Technology, University Post, Kumasi, Ashanti Region, Ghana

## Abstract

The rapid emergence and spread of antimicrobial resistance has become a global public health concern that threatens the effective treatment of infectious diseases. One major approach adopted to overcome antimicrobial resistance is the use of plant extracts individually and/or with combination of antibiotics with plant extracts, which may lead to new ways of treating infectious diseases and essentially representing a potential area for further future investigations. In this study, the antifungal activities of *Azadirachta indica* leaf and *Catharanthus roseus* flower extracts against fluconazole-resistant *Candida albicans* strains (isolated from pregnant women with vulvovaginal candidiasis) and anti-methicillin-resistant *Staphylococcus aureus* (MRSA) were evaluated by agar well diffusion, microdilution, and biofilm inhibition assays. Subsequently, the determination of the combined antimicrobial activity of the individual plant extracts with (fluconazole and voriconazole) and (ampicillin, tetracycline, and streptomycin) against *C. albicans* strains and MRSA, respectively, was evaluated by checkerboard microdilution assay. Results from the study showed that the antimicrobial activity of the two plant extracts determined by time-kill kinetics was fungistatic with their MICs ranging from 0.1 to 4 mg/mL. Interestingly, all extracts were proved as good biofilm inhibitors of resistant *C. albicans* and MRSA from 10.1 to 98.82%. Their combination interaction with fluconazole, voriconazole, ampicillin, tetracycline, and streptomycin ranged from synergy to antagonism as per the parameters used. Overall, these results showed that *A. indica* leaf and *C. roseus* flower extracts have significant antifungal property. Furthermore, *A. indica* leaf and *C. roseus* flower extracts alone or in combination with fluconazole and voriconazole could provide a promising approach to the management of candidiasis caused by drug-resistant strains as well as their interaction with the antibacterial agents to combat the common infections caused by MRSA.

## 1. Introduction

Antimicrobial resistance is of grave concern globally since it threatens the gains made over the years to reduce the prevalence of infectious diseases, most especially in Africa [1]. It is responsible for about 700,000 deaths worldwide with a greater percentage of this death happening in low- and middle-income countries [2]. The development of resistance mechanisms such as biofilm formation, efflux pump possession, target site diversion, and resistance gene acquisition among others by bacterial species to inactivate the effectiveness of most antibacterial agents has become a major health security threat to the medical world in recent times [2, 3]. Among these mechanisms, biofilms have been noted to form 65% of microbial infections and bacteria living in them develop resistance to antibiotics a thousand times than those existing as planktonic cells, thereby calling for urgent attention [4]. Recent studies have proven that about 95% of *Staphylococcus aureus* are now resistant to penicillin as well as methicillin given to patients in hospitals and communities [5]. Methicillin-resistant *S. aureus* (MRSA) is one of the common causes of multidrug resistance infections with significant morbidity and mortality [6]. This has been attributed to the abuse of over-the-counter (OTC) medications most especially without prescription [7]. In many clinical settings, recurrent candidiasis has been treated mostly by administering standard antifungal drugs, such as azoles, polyenes, and echinocandins. However, in recent times, reports have shown that there has been an increase in the number of resistant *Candida* species especially *C. albicans* against most of these standard drugs [8, 9].

The resistance of *Candida* species to many of these antifungal agents has been attributed to the formation of *Candida* biofilms, which can occur on mucosa or endothelia surfaces as well as medical devices such as indwelling joint prostheses, cardiac valves, and intravascular and urinary catheters [10, 11].

As such, the intervention adopted to combat this menace has been the use of combination therapy either with orthodox medications or plant-based products. Because these combination drugs have been shown to have the ability to modulate the resistance capacity of these microbial organisms [12, 13]. Despite these gains in the fight against antibiotic resistance, there still remains much more to be done in search of new drugs which are efficacious to help curb the ever-increasing antimicrobial resistance menace. Natural products and their plant derived compounds are mostly a source of new drug agents as well as lead compounds with little or no side effects [14]. According to the WHO, about 65% of the world's population rely on the use of herbs for their primary health care needs most especially in the sub-Saharan Africa region [15]. *Azadirachta indica* and *Catharanthus roseus* are two plants that possess antimicrobial activities are used traditionally for various infectious disease conditions in Ghana.

Neem plant (*Azadirachta indica* A. Juss) is a tree belonging to the family Meliaceae. The different parts of the plant are used as antiseptic, diuretic, and in the management of diseases such as, cough, nausea, vomiting, fever, and peptic ulcer [16]. *Catharanthus roseus* (L. G. Don) from the family Apocynaceae contributes vastly to the treatment of several diseases including cancer. It is an evergreen herbaceous plant native to Madagascar [17]. A study on this plant has shown that the ethanol leaves extract of *C. roseus* showed antimicrobial activity against *Staphylococcus aureus, Escherichia coli*, and *C. albicans* [18, 19].

This study therefore sought to investigate the antimicrobial, antibiofilm, and resistance modulatory potentials of *C. roseus* and *A. indica* on biofilm forming fluconazole-resistant *C. albicans* and MRSA.

## 2. Methods

### 2.1. Experimental Plant Materials

The plant materials *A. indica* leaves and *C. roseus* flowers were collected from the campus of the University of Health and Allied Sciences, Ho Municipality. The plants were identified, and voucher specimens were deposited in the Herbarium of the Institute of Traditional and Alternative Medicine, University of Health and Allied Sciences, Ho, Ghana, with a voucher number (UHAS/ITAM/2020/L002 and UHAS/ITAM/2020/L003).

### 2.2. Test Organisms

Four fluconazole-resistant *Candida albicans* isolates were obtained from the Department of Medical Microbiology, Ho teaching Hospital, Ghana. These strains were primarily isolated from pregnant women with vulvovaginal candidiasis. To guarantee the purity of the *Candida* isolates, separate yeast colonies were subcultured on Sabouraud Dextrose Agar (SDA) (Oxoid Ltd., Hampshire, UK), with chloramphenicol and incubation at 37°C for 24–48 h. Subsequently, presumptive identification of *C. albicans* was carried out by examination of colony morphology, microscopic examination of Gram-stained preparations, production of chlamydospore, germ tube test, and sugar fermentation tests [20]. Confirmation with API ID 32C strips was done using standard microbiological procedures (BioMerieux, France). Additionally, methicillin-resistant *Staphylococcus aureus* (NCTC 29212) used in this study was obtained from the Microbiology Laboratory, Department of Biomedical Sciences, School of Basic and Biomedical Sciences, UHAS, on the basis of its implication in most infections.

### 2.3. Detection of Susceptibility to Fluconazole

Antifungal susceptibility testing of fluconazole (25 *μ*g) (Oxoid Ltd., Basingstoke, UK) was carried out by disc diffusion method as described by Clinical and Laboratory Standards Institute (CLSI, document M44-A2, 2009). A zone diameter of ≥19 mm was considered sensitive, 15 to 18 mm was considered dose-dependently susceptible, and a diameter ≤14 mm was considered resistant [21].

### 2.4. Extraction and Preparation of Plant Materials

Each of the plant parts of *A. indica* and *C. roseus* washed under running tap water were air-dried at room temperature (25 to 32°C) for one to two weeks [22]. The dried plant materials were milled using a laboratory warring blender into coarse powder. Each processed plant material (40 g) was cold macerated with 200 mL of 70% v/v ethanol for two days with intermittent stirring. The mixture was then filtered with a Whatman paper No. 1 and filtrate was evaporated to dryness using rotary evaporator under reduced pressure at 38°C, oven-dried at 40°C, and stored in a fridge at 4°C until use.

### 2.5. Phytochemical Screening

Presence of alkaloids, flavonoids, steroids, terpenoids, saponins, tannins, and glycosides were tested as per the method described by Visweswari et al. [23] and Neglo et al. [24] with slight modification. Grading of the final reaction of the secondary metabolites was done by comparing the results obtained for the plant extracts using how deep or light the color change was seen.  Table 1 shows a summary of the various tests that were conducted.

### 2.6. Determination of Antifungal Activity of the Plant Materials

The antifungal activity of the extracts was determined using both the Kirby–Bauer agar well diffusion method and the broth microdilution method [25].

### 2.7. Agar Well Diffusion

20 mL of sterile Muller–Hinton agar was poured and allowed to set and then inoculated with 100 *μ*l of 1x 10^6^ colony-forming units (CFU)/mL of MRSA. All the strains (MRSA and *C. albicans*) were cultured overnight and grown at 37°C in Muller–Hinton broth with further dilution to 0.5 McFarland standards with saline and then inoculated on Muller–Hinton agar. Five wells were bored in each plate using a cork borer (No. 3, 5 mm). These wells were filled with 100 *μ*l of 10, 20, and 40% of ethanol extracts of the two plant materials. 20% DMSO was used as a negative control, whereas tetracycline (10 *μ*g/disc) and voriconazole (25 *μ*g/disc) were used as positive control for MRSA and *C. albicans* inoculum, respectively. Each of the extracts were then allowed to diffuse for 15 mins at room temperature after which they are incubated at 37°C for 48 h and zones of inhibitions were recorded. The procedure was performed in triplicate.

### 2.8. Minimum Inhibitory Concentrations of the Extracts and the Standard Antibiotics/Antifungal Agents

The minimum inhibitory concentrations (MICs) of the extracts and the referenced drugs (ampicillin, tetracycline, and streptomycin fluconazole and voriconazole dissolved in sterile distilled water except tetracycline in 10% ethanol) were carried out according to the method described by [25] with slight modification.

Briefly, each well of a 96-well microtitre plates was filled with 150 *μ*L of the double-strength Mueller–Hinton broth. This was followed by dilutions of each of the extracts and the referenced drugs ranging from 0.063 to 256 mg/mL and 0.063 to 128 *μ*g/mL by adding 150 *μ*L of each test sample for both the extracts and the drugs, respectively. In all cases, one well served as positive control inoculated with each test microorganism and the broth stock in the test tube as the negative control without organism. Afterwards, 150 *μ*L of 10^6^ cfu/mL of each test microorganisms prepared in the broth was added to each well.

The microtitre plates were then subjected to incubation at 37°C for 24 hours, after which 40 *μ*L of 3-(4,5- dimethylthiazole-2- yl)-2,5-diphenyltetrazolium bromide (MTT) (0.2% w/v) was added to each well. The plate was incubated at 37°C for 30 min, and the appearance of purple color signified growth. The concentration at which the extracts and the referenced drugs did not show any change in color was noted as the minimum inhibitory concentration.

### 2.9. Determination of Synergistic Effect of Test Plant Samples and Selected Antimicrobial Agents

*In-vitro* analysis of the interaction between test samples from *A. indica* and *C. roseus* and (i) antibiotics (ampicillin, tetracycline, and streptomycin) against MRSA (NCTC 19243) and (ii) antifungals (fluconazole and voriconazole) against *C. albicans* strains were evaluated by adopting checkerboard microdilution assay as described previously by Khodavandi et al. [26] and Dickson et al. [27] with slight modifications. The tested concentrations for each ampicillin, tetracycline, streptomycin, fluconazole, and voriconazole and 0.5 mg/ml subinhibitory concentrations of each test plant sample ranged from 0.063 to 64 *μ*g/mL.

The mode of the interactions was measured by calculating the fraction inhibitory concentration index (FICI). The FICI was estimated by

(1) $$\begin{array}{c} \text{FICI}=\frac{Ac}{Aa}+\frac{Ec}{Ea}, \end{array}$$

where *Ac* is the MIC of antibiotic/antifungal in combination, *Ec* is the MIC of each test plant sample in combination, *Aa* is the MIC of each antibiotic/antifungal alone, and *Ea* is the MIC of each test plant sample alone. The interaction was considered synergistic if the FICI was ≤0.5, partial synergistic if FICI was >0.5 and <1, additive if FICI was = 1, no difference if the FICI was >1 and ≤4, and antagonistic if the FICI was >4.0.

### 2.10. Formation of Biofilms by the Clinical *C. albicans* Strains and MRSA (NCTC 19243)

The biofilm-forming potentials of the clinical *C. albicans* cultures and clinical standard MRSA (NCTC 19243) diluted to 0.5 McFarland standard in Mueller–Hinton broth were employed per the protocol described by Haque et al. [28] and Neglo et al. [24] with slight modification. Briefly, 200 *μ*L of each of the standardized inoculum prepared was added to 2 mL of broth in test tubes and incubated at 37°C for 24 and 48 hours with regard to bacteria or fungi, respectively.

Planktonic cells were aspirated and washed from the tubes to get rid of floating cells; the tubes were then dried at 25–28°C in incubator and stained with 2 mL of 0.1% crystal violet for 15 mins after which they were washed with sterile water and further dried at room temperature. The adherent microbial biofilms on the walls of the tubes were reconstituted with 2 mL ethanol and the absorbance of each sample was read at 595 nm with a UV spectrophotometer (Jenway, Bibby Scientific Ltd., Stone, Staffordshire, UK).

The optical density (OD) of the sterile broth was subtracted from that of the microbial biofilms formed to compensate for the background absorbance. Each of the procedures was performed in triplicate.

### 2.11. Biofilm Inhibition Effects of the Extracts

The potential of the various extracts to inhibit biofilm formation was determined as per the protocol described by Haque et al. [28] and Alshami and Alharbi [29]. One ml of each of the extracts was diluted with 1 ml of the Mueller–Hinton broth in sets of test tubes arranged to arrive at concentrations ranging from 32 mg/ml to 1 mg/ml, after which 10 *μ*l of each of the standardized MRSA containing 10^5^ per mL microorganisms was added. Control wells containing no extracts were included as well.

Each of the tubes was then incubated undisturbed at 37°C for 48 hours. The planktonic cells were aspirated and washed from the tubes and subsequently dried at 25–28°C in incubator, after which they were stained with 1 mL of 0.1% crystal violet for 15 mins. Each tube was further washed with sterile water and dried at room temperature. The adherent microbial biofilms on the walls of each tubes were reconstituted with 1 mL ethanol and the absorbance of each sample read at 595 nm with a UV spectrophotometer (Jenway, Bibby Scientific Ltd., Stone, Staffordshire, UK) after being blanked, and the optical density of the culture media control was subtracted to obtain inhibitory effects of the extracts. Each of the procedures was performed in triplicate.

The biofilm inhibition potential of each of the extracts to reduce the optical density compared to the negative control was noted as the biofilm inhibitory activity.

(2) $$\begin{array}{c} \;\%\text{ biofilm inhibition:optical density }\left(OD\right)\text{ of control}-\;\frac{OD\text{ of treatment}}{OD\text{ of control}}\times 100. \end{array}$$

### 2.12. Time-Kill Kinetics Assay of the Extracts

The time-kill kinetics of the various extracts were carried as per the protocol designed by Appiah et al. [30] with slight modification. Briefly, the microbial strain MRSA (NCTC 19243) and clinical *C. albicans* strains were standardized to 10^6^ cfu/mL cell concentration in test tubes. Subsequently, concentrations equal to the MIC, twice the MIC, four times and 8 times MIC of each extract as in Tables 2 and 3 were prepared and mixed with sterile broth in test tubes. Each of the tested microorganisms is then added and incubated at 37°C. Aliquots of 1 ml of each of the various medium were pipetted into well-labelled Petri dishes at intervals of 0, 6, 18, 30, 54, and 72 h for the individual tested organisms. Nutrient agar was then poured onto each medium transferred and incubated at 37°C for 24 h. Inoculums of each of the strains were treated similarly alongside as the control. The colony-forming unit of the test organisms was determined and the experiment was carried out in triplicate for each. Graphs of log CFU were plotted against time for each treatment and the data obtained from the study was analysed using one-way ANOVA followed by post hoc test using Graph Pad Prism Version 5.0.q.

### 2.13. Statistical Analysis

All measurements are expressed as mean ± standard error mean (SEM) or mean ± SD of independent experiments. IC_50_ values were obtained by interpolations from standard curves. All tests were carried out in triplicate.

## 3. Results

### 3.1. Preliminary Phytochemical of the Extracts

The phytochemical results obtained for the ethanolic extracts are presented in Table 4. The alkaloids, reducing sugars, and saponins are present in all the extracts in varied proportions while the flavonoids are absent in all the extracts. However, *A. indica* leaf and *C. roseus* flower extracts possessed a high-to-moderate level of the alkaloids with steroids being absent in them. *A. indica* leaf and *C. roseus* leaf extracts had high-to-low levels of reducing sugars; the tannins were low in *A. indica* leaf and absent in the other two extracts.

### 3.2. Antimicrobial Activity of Plant Extracts

The ethanol extracts of both *A. indica* and *C. roseus* had antimicrobial activities against all microbes employed in this study. At various concentrations (40, 20, and 10%), each of the extracts recorded significant antifungal activity against all the fluconazole-resistant *C. albicans* strains and MRSA (NCTC 12493) in the disc diffusion assay with varied zones of inhibition (Table 5). *A. indica* extract had the higher inhibitory activity against MRSA with a value of 19.33 ± 0.67 mm at 40% as compared to *C. roseus.*

Similarly, *A. indica* recorded the highest inhibitory activity against all *C. albicans* strains, out of which CA 4 gave the highest value of 19.33 ± 0.33 at 40%, while *C. roseus* showed the least activity in all varied concentration, except against CA 3. Both controls, positive and negative, however showed no activity, showing the relevant efficacy of the test extracts.

### 3.3. MIC of Plant Extracts and Antimicrobial Agents

In the broth dilution assay, microbes in inoculums were reduced in a dose-dependent manner by the extracts (Table 6). The trend of activity was however in the same order as in the disc diffusion method. While both plant extracts had good MICs against all the fluconazole-resistant *C. albicans* strains, the MICs of the extracts varied from one *C. albicans* strain to another within the range 0.1–4 mg/mL. With the test antifungal agents, the MICs ranged from 4 to 16 *μ*g/mL as shown in Table 6. Similarly, both plant extracts had good MICs against MRSA (NCTC 12493) at 1 mg/L, while the test antibiotic's MICs ranged from 8 to 32 *μ*g/mL (Table 6).

### 3.4. Synergistic Effect of Test Plant Samples and Selected Antimicrobial Agents against MRSA

The interactions of the antifungal combinations of the extracts of *A. indica* and *C. roseus* with fluconazole and voriconazole as well as ampicillin, tetracycline, and streptomycin examined using slightly modified microdilution checkerboard method are summarised in Tables 2 and 3. *A. indica* exhibited a significant synergy with fluconazole against a resistant strain of *C. albicans* 1 and 4, then addictive interaction against *C. albicans* 3 and antagonism against *C. albicans* 2. On other hand, *A. indica* shows a partial synergy with voriconazole against *C. albicans* 4, addictive interaction against *C. albicans* 3, no difference in interaction against *C. albicans* 2 and antagonism interaction against *C. albicans* 1. Again, against MRSA, there was a synergy between ampicillin and tetracycline with *A. indica* and no difference in interaction with streptomycin. Concurrently from the study, while *C. roseus* with fluconazole demonstrated partial synergy against *C. albicans* 4, addictive against *C. albicans* 2, no difference against *C. albicans* 3, and antagonism against *C. albicans* 1 in combination with voriconazole as well, same synergistic antifungal activities were recorded against the *C. albicans* strains. Again, in the combination of *C. roseus* with ampicillin, tetracycline, and streptomycin against MRSA, there was antagonism, partial synergy, and no difference interactions noted, respectively.

### 3.5. Biofilm Inhibition Potential of Plant Extracts

**Figure 1:**
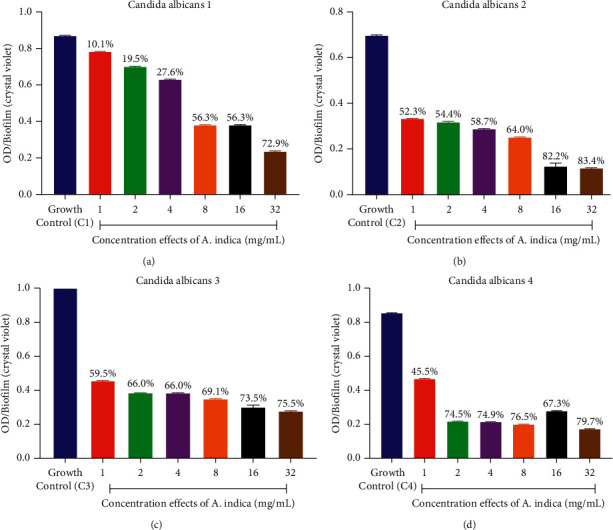
(a–d) Graphs showing the effect of different concentrations of ethanolic extracts of *A. indica* on the amount of biofilm formed (optical density (OD)) by fluconazole-resistant *C. albicans* strains.

**Figure 2:**
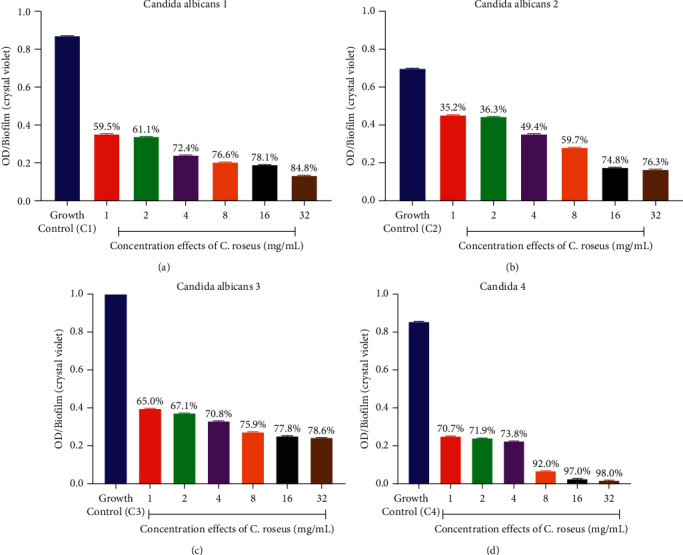
(a–d) Graphs showing the effect of different concentrations of ethanolic extracts of *C. roseus* on the amount of biofilm formed (optical density (OD)) by fluconazole-resistant *C. albicans* strains.

**Figure 3:**
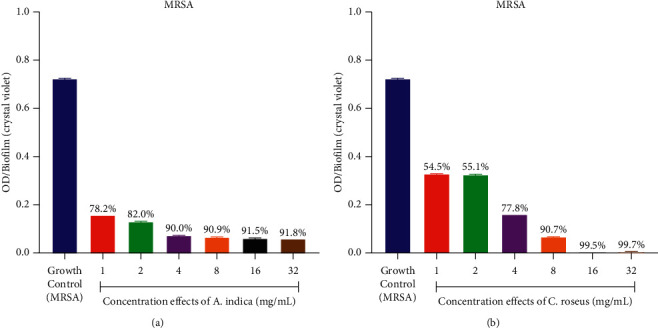
(a-b) Graphs showing the effect of different concentrations of ethanolic extracts of *A. indica* and *C. roseus* on the amount of biofilm formed (optical density (OD)) by MRSA (NCTC12493).

The result obtained showed that each *C. albicans* strains possessed considerable amount of biofilm with their absorbances ranging from 0.858 ± 0.001 to 1.102 ± 0.001 with the summary of the biofilm inhibition as shown in Figures 1 and 2. The effect of different concentrations ranging from 1 to 32 mg/ml of each of the extracts on the *C. albicans* biofilms established the reduction of biofilm by various extracts. Biofilm formed in the absence of the extracts was used as negative control. The biofilm inhibition potentials of the *A. indica* extract varied from 10.1 to 83.4% with the highest IC_50_ of 1.57 ± 0.01 mg/mL against *C. albicans* 4 (Figure 1, Table 7), while that of the *C. roseus* varied from 35.2 to 98.82% with the best IC_50_ of 1.29 ± 0.01 mg/mL against *C. albicans* 4 (Figure 2, Table 7). All the *C. albicans* strains showed reduced biofilm formation in the presence of different concentrations of the test extracts. The MRSA strain also showed reduced biofilm formation in the presence of different concentrations of the test extracts (Figure 3, Table 7). Biofilm formation was determined by crystal violet staining. Values are means of three independent experiments. *P* < 0.05 denotes between growth control and different concentrations of the extracts.

### 3.6. Time-Kill Kinetics Assay of Ethanolic Extracts of *A. indica* and *C. roseus* against Fluconazole-Resistant Strains of *Candida albicans*

**Figure 4:**
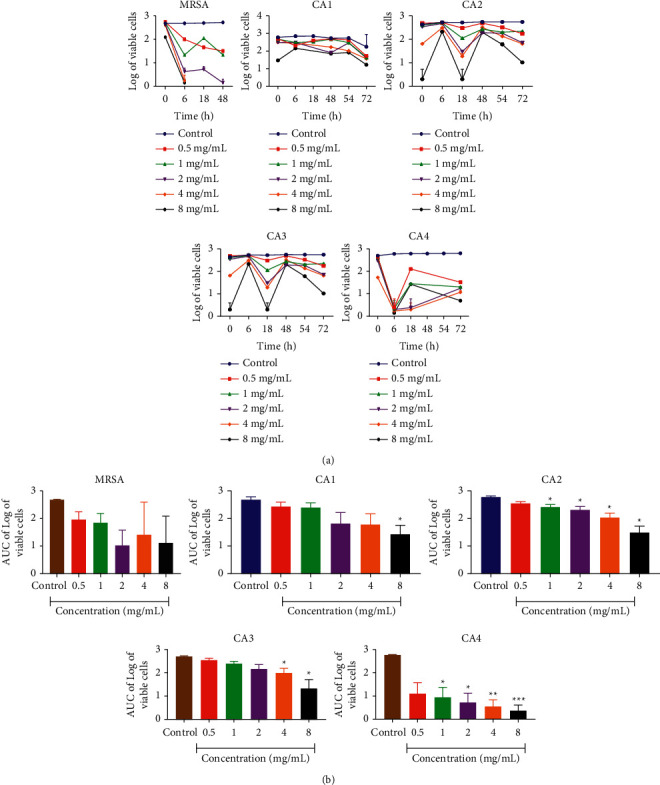
Time-kill kinetics of *A. indica* ethanolic extract against fluconazole-resistant *C. albicans* strains and MRSA (NCTC12493). (a) Time-kill kinetics curve and (b) AUC of time-kill kinetics. *n* = 5; values are mean ± SEM. ^*∗*^^,^^*∗∗*^*p* < 0.0001 (one-way ANOVA followed by Dunnett's post hoc test); AUC: area under the curve, CA1: *C. albicans* 1, CA2: *C. albicans* 2, CA3: *C. albicans* 3, and CA4: *C. albicans* 4.

**Figure 5:**
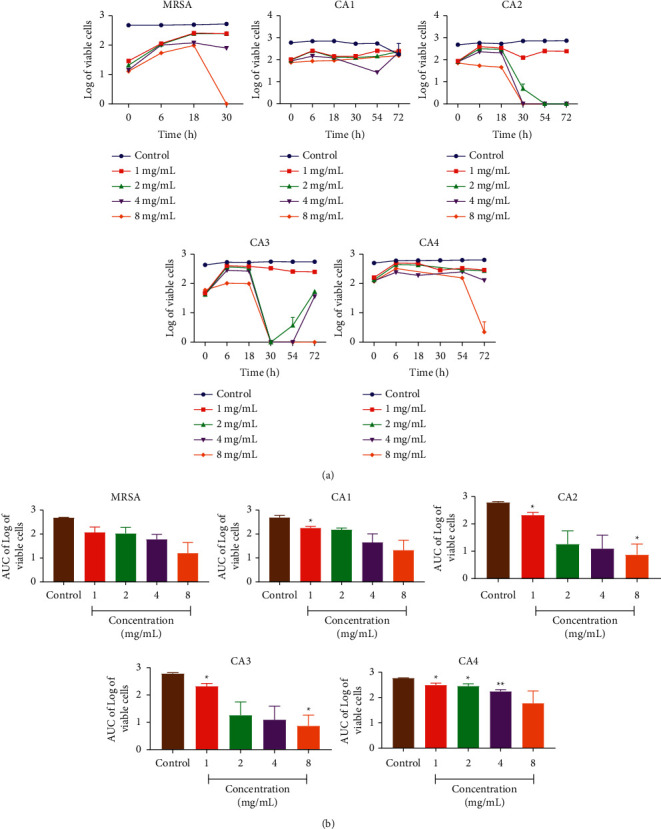
Time-kill kinetics of *C. roseus* ethanolic extract against fluconazole-resistant *C. albicans* strains and MRSA (NCTC12493). (a)Time-kill kinetics curve and (b) AUC of time-kill kinetics. *n* = 5; values are mean ± SEM. ^*∗*^^,^^*∗∗*^*p* < 0.0001 (one-way ANOVA followed by Dunnett's post hoc test); AUC: area under the curve, CA1: *C. albicans* 1, CA2: *C. albicans* 2, CA3: *C. albicans* 3, and CA4: *C. albicans* 4.

Time-kill curves were performed for the resistant *C. albicans* strains using different concentrations of *A. indica* and *C. roseus* extracts with the MIC values ranging from 0.5 to 8 times. The results obtained for the time-kill curves are summarised in Figures 4 and 5 for each, respectively. The effects against the *C. albicans* strains were fungistatic (*P* < 0.05). Fungistatic activity has been defined as <3 log reduction in CFU/mL using time-kill.

The present study allows us to establish that *A. indica* leaf and *C. roseus* flower extracts has a fungistatic effect against all the *Candida albicans* strains used.

## 4. Discussion

Antimicrobial agents have been used in contemporary medicine to help fight microbial infections [31]. However, the emergence and spread of antimicrobial resistance (AMR) threatens the effective control and management of various microbial infections worldwide [31]. The progress made in reducing mortality and morbidity as a result of early use of antibiotics based on empiric guidelines is under serious threat if steps are not taken to curb the menace of AMR [32]. Methicillin-resistant *Staphylococcus aureus* (MRSA) and *Candida albicans* are microbial organisms which cause several infectious diseases and have proven to be resistant to commonly used antimicrobial agents. Several mechanisms have been postulated to be responsible for microbial organisms' ability to resistant antimicrobial agents. One of such mechanisms is the formation of biofilm, which is a complex structure of microbiome with either different bacterial colonies or single type of cells, which tend to adhere to surfaces [33].

In fact, many research scientists are in the business of looking for novel compounds which are effective against several microbial organisms responsible for common infectious diseases and has little or no side effect. This study investigated the antimicrobial and antibiofilm effect of extracts of *C. roseus* and *A. indica* and their possible pharmacokinetic interactions with already known antimicrobial agents.

In the antimicrobial study, all the extracts significantly inhibited the growth of MRSA at the concentrations used. *C. roseus* has been reported by Shil et al. [34] to have antimicrobial property against *S. aureus*, confirming the findings of this study. The results on the antimicrobial activity of *A. indica* extract corroborate with studies done by Quelemes et al. [35] where they concluded that ethanolic leaf extract of *A. indica* inhibited the growth of MRSA. Several research works have shown that plants contain certain phytochemical constituents, which are responsible for their antimicrobial property. A review by Othman et al. [36] shows that alkaloids and polyphenols are largely responsible for plants' antimicrobial properties. These constituents were found in the two extracts used and hence may be responsible for the antimicrobial effect seen in this study.

The *Candida albicans* (CA) strains used in the antifungal experiment were clinical isolates from pregnant women. They were obtained from pregnant women because a work done by Masri et al. [37] revealed that pregnant women are a great source of CA and hence they are our choice for the source of the CA strains. All the extracts inhibited the growth of all the strains of CA used in this experiment indicating their antifungal property. This result was in tandem with some previous studies where they reported that various extracts of *A. indica* and *C. roseus* inhibited the growth of CA in their respective experiments [38, 39]. It is worthy of note that the standard drugs used, that is, fluconazole and voriconazole, which are triazole antifungal agents, could not inhibit the growth of all the CA strains used, confirming that the strains used were fluconazole-resistant.

One of the mechanisms used by microbial organisms to resist the effect of drugs is the formation of biofilm [40]. Bacteria in biofilm are surrounded by an extracellular matrix, which may physically prevent the penetration of antimicrobial agent through the cell wall of the microbial organism [40]. Ideally, antibiofilm property is an important trait that is exhibited by new antimicrobial agents. In this experiment, the ability of MRSA and CA to produce biofilms as a protective measure was established. However, simultaneous culturing of the microbial organisms and the various extracts showed a significant inhibition of the biofilm in all the strains used in this experiment indicating the antibiofilm property of both *A. indica* and *C. roseus* extracts. This corroborated with the earlier studies where they revealed that *A. indica* inhibited biofilm formation of *Pseudomonas aeruginosa* whereas *C. roseus* inhibited biofilm formation by *P. aeruginosa* and *S. aureus*, respectively [41, 42]. The ability of the extracts to inhibit biofilm formation could be attributed to the fact that their phytoconstituents could possibly destroy the structure of the microbial organism and also prevent the synthesis of peptidoglycan [43]. This result indicates that these extracts could be used in the management of microbial infections, which are resistant to conventional antimicrobial agents due to their ability to produce biofilms [14].

In the management of a number of infectious diseases, two or more drugs may however be employed. The therapeutic efficacy of the drug combination depends on the interactions of drugs combined. Synergistic combinations help to reduce emergence of resistant mutants and toxicity, exhibit more antimicrobial activity, and are more effective against mixed infections [44]. It is for this reason that an attempt was made in this study to ascertain potential pharmacodynamic interactions between the various concentration of the extracts and the standard drugs used against the experimental microbial organisms.

In the antifungal experiment, a combination of either *A. indica* or *C. roseus* extract and fluconazole or voriconazole showed several pharmacodynamic interactions on the different strains of the CA used. These interactions include antagonism, synergism, and additive and partial synergism. The difference in the herb drug interactions despite the fact that constant concentrations of both extracts and standard drugs used may be as a result of the difference in the strains of *C. albicans* used since they were obtained from different subjects. This interesting finding brings back the topic of pharmacogenomics where scientists are of the view that an individual's genetic make-up influences his or her response to drug therapy [45].

With regard to the herb drug interaction in the MRSA experiment, a combination of *A. indica* (AI) with either ampicillin or tetracycline showed a synergistic relationship, whereas antagonism was revealed in the combination of AI and streptomycin. This result however was opposite to the findings made by Ngwu et al. [46] where there was rather an antagonism observed with AI and tetracycline and synergism observed with AI and streptomycin. This observation may be due to the difference in the source of AI used since it is believed that regional and climate difference may affect active constituents found in medicinal plants [36]. With regard to *C. roseus* (CR) and ampicillin combination, there was an antagonistic interaction whilst there was partial synergy in the tetracycline and CR combination. This result was in contrast with that recorded by Shil et al. [34] where CR had a synergistic interaction with ampicillin. The difference in results may be attributed to the organism used; whilst they used multiple drug-resistant *S. aureus* strain, in this experiment MRSA was used. Again, different climate conditions could also affect the phytoconstituents in the CR used for the two studies.

Time-kill kinetics assay is used to study the activity of an antimicrobial agent against microorganisms and to further categorize them into either bactericidal and or fungicidal or bacteriostatic and or fungistatic. In this experiment, AI and CR were bacteriostatic and fungistatic, respectively.

It could be concluded that *A. indica* and *C. roseus* extracts possess interesting antimicrobial and antibiofilm activity and have the potential to have various pharmacodynamic interactions with standard antimicrobial agents.

## 5. Conclusion

The discovery of new and effective natural bioactive compounds with high antifungal and anti-MRSA activities, specifically biofilm-forming cells, will signify a substantial impact on the treatment and management of *C. albicans* infections as well as other related bacterial infections. Our results therefore indicated that the extracts of *A. indica* leaf and *C. roseus* flower alone or in combination with the test antifungals could provide a promising means of the management of vulvovaginal candidiasis caused by drug-resistant strains as well as when in combination with the selected antibacterial agents against recurrent infections caused by the MRSA. However, additional researches are required to identify the antimicrobial activity of *A. indica* leaf and *C. roseus* flower and its bioactive elements against *C. albicans* and non-*C. albicans* species implicated in different clinical infections, not only vulvovaginal candidiasis as well as other resistant bacterial strains aside MRSA.

## Figures and Tables

**Table 1:** Phytochemical screening methods as described by Visweswari et al. [23] and Neglo et al. [24].

Phytochemical	Test	Observation
Alkaloid	Wagner's reagent (I_2_/KI) was used. Minute quantity of extracts was dissolved in dilute HCl and filtered. Few drops of Wagner's reagent (I_2_/KI) were added to about 2 mL of the filtrate.	Formation of brownish/red precipitate was used to determine the presence of alkaloid.

Flavonoids	Sulphuric acid (H_2_SO_4_) test was done by treating a fraction of the extract with concentrated H_2_SO_4._	Formation of orange color was used to detect the presence or absence of flavonoids.

Steroids	Liebermann–Burchard test was used. Four milligrams of the extracts was treated with 0.5 ml of acetic anhydride and 0.5 mL of acetic acid. Concentrated H_2_SO_4_ was slowly added.	The development of a reddish/brown color indicated the presence of steroids.

Terpenoids	Liebermann–Burchard test was used. Four milligrams of the extracts was treated with 0.5 ml of acetic anhydride and 0.5 mL of acetic acid. Concentrated H_2_SO_4_ was slowly added.	The development of a blue-green color indicated the presence of terpenoids.

Saponins	This was tested for using foam test. Exactly 0.5 g of the plant extract was dissolved in 2.5 mL of distilled water. The mixture was shaken vigourously.	The presence of foam indicated the presence of saponins.

Tannins	Ferric chloride test was used to test for the presence of tannins. An exact amount of 0.5 g of the extract was boiled in 20 mL of distilled water and filtered afterwards. Few drops of 0.1% of FeCl_3_ were added.	The presence of brownish-green, brownish-black, or blue-black color was used to detect the presence of tannins.

Glycosides	Benedict's test was used for the detection of glycosides. Precisely, 0.5 g of plant extract was dissolved in 5 ml of distilled water. Exactly 2 mL of Benedict's solution was heated and 8 drops of the dissolved sample were added and allowed to boil for 5 minutes.	Formation of brick-red precipitate indicated the presence of glycosides.

**Table 2:** Synergistic effect of *A. indica* and *C. roseus* on fluconazole and voriconazole against resistant *C. albicans* strains.

Test sample	Fluconazole	Interpretation	Voriconazole	Interpretation
	FIC index		FIC index	
*A. indica*				
CA1	0.13	Synergy	16.13	Antagonism
CA2	5.00	Antagonism	1.5	No difference
CA3	1.00	Additive	1.00	Additive
CA4	0.13	Synergy	0.63	Partial synergy

*C. roseus*				
CA1	5.25	Antagonism	37.00	Antagonism
CA2	1.00	Additive	1.00	Additive
CA3	2.07	No difference	2.17	No difference
CA4	0.63	Partial synergy	0.63	Partial synergy

**Table 3:** Synergistic effect of *A. indica* and *C. roseus* on selected antibacterial agents against MRSA.

Test sample	Ampicillin	Interpretation	Tetracycline	Interpretation	Streptomycin	Interpretation
	FIC index		FIC index		FIC index	
*A. indica*						
MRSA	0.500	Synergy	0.500	Synergy	2.50	No difference
*C. roseus*						
MRSA	8.50	Antagonism	0.75	Partial synergy	2.50	No difference

**Table 4:** Phytochemical screening results of ethanolic extracts of *Azadirachta indica* and *Catharanthus roseus.*

Phytochemical	Presence	
	*A. indica* leaves	*C. roseus* flowers
Alkaloids	+	+
Flavonoids	−	−
Steroids	−	−
Terpenoids	−	+
Saponins	+	+
Tannins	+	−
Reducing sugars	+	+

Note: +: present; −: absent.

**Table 5:** Diameter of inhibition zone of ethanolic extracts of *A. indica* and *C. roseus* against clinical isolates fluconazole-resistant *C. albicans* strains and MRSA (NCTC 12493).

Plants (ethanol extracts)	Conc. (w/v %)	Zone of inhibition (mm) (mean ± SEM)				
		CA1	CA2	CA3	CA4	MRSA (NCTC12493)
AI	40	17.33 ± 0.33	15.67 ± 0.33	15.33 ± 0.33	19.33 ± 0.33	19.333 ± 0.67
	20	13.66 ± 0.33	11.67 ± 0.33	13.67 ± 0.33	13.00 ± 0.578	13.000 ± 0.58
	10	10.33 ± 0.33	6.33 ± 0.33	10.67 ± 0.33	10.67 ± 0.33	10.667 ± 0.33
CR	40	16.00 ± 0.57	13.0 ± 0.58	16.67 ± 0.33	14.33 ± 0.333	17.667 ± 0.33
	20	11.667 ± 0.33	10.67 ± 0.33	14.33 ± 0.33	12.33 ± 0.333	11.000 ± 0.58
	10	0.000 ± 0.00	9.33 ± 0.33	11.33 ± 0.33	9.67 ± 0.333	6.667 ± 0.67
Positive control (fluconazole, 25 *μ*g)		NI	NI	NI	NI	—
D/control (tetracycline, 10 *μ*g)	—	—	—	—	—	27.33 ± 1.200
Negative control (20% DMSO)		NI	NI	NI	NI	NI

Values are shown in triplicate and represented as mean ± SEM. AI: *Azadirachta indica*, CR: *Catharanthus roseus*, CA1: *Candida albicans* 1, CA2: *Candida albicans* 2, CA3: *Candida albicans* 3, CA4: *Candida albicans* 4, and NI: no inhibition.

**Table 6:** Minimum inhibitory concentration of ethanolic extracts and antimicrobial agents against fluconazole-resistant *C. albicans* strains and MRSA (NCTC12493).

*MIC of plant ethanol extracts against test organisms*					
Test organisms	AI (mg/L)	Test organisms		CR (mg/L)	

CA 1	4.0	CA 1		0.1	
CA 2	0.1	CA 2		1.0	
CA 3	0.5	CA3		0.3	
CA 4	4	CA 4		4.0	
MRSA	1.0	MRSA		1.0	

*MIC of antimicrobial agents against test organisms*					
Test organisms	Fluconazole (*μ*g/mL)	Voriconazole (*μ*g/mL)	Tetracycline (*μ*g/mL)	Ampicillin (*μ*g/mL)	Streptomycin (*μ*g/mL)

CA 1	>64	4.0	NE	NE	NE
CA 2	>64	16.0	NE	NE	NE
CA 3	>64	8.0	NE	NE	>64
CA 4	>64	4.0	NE	NE	NE
MRSA	NE	NE	32.0	16.0	8.0

AI: *Azadirachta indica*, CR: *Catharanthus roseus*, CA1: *Candida albicans* 1, CA2: *Candida albicans* 2, CA3: *Candida albicans* 3, CA4: *Candida albicans* 4, MIC: minimum inhibitory concentration, and NE: not evaluated.

**Table 7:** IC_50_ values of biofilm inhibition by ethanolic extracts of *A. indica* and *C. roseus* against fluconazole-resistant *C. albicans* strains and MRSA (NCTC12493).

*A. indica*		*C. roseus*	
Test organisms	IC_50_ values (mg/mL)	Test organisms	IC_50_ values (mg/mL)
CA1	8.87 ± 0.10	CA1	1.64 ± 0.01
CA2	2.39 ± 0.02	CA2	3.81 ± 0.06
CA3	1.69 ± 0.01	CA3	1.50 ± 0.01
CA4	1.57 ± 0.01	CA4	1.29 ± 0.01
MRSA (NCTC12493)	1.002 ± 0.001	MRSA	1.73 ± 0.002
Control	—	Control	—

Each value is the average of three independent experiments ± SDs. CA1: *Candida albicans* 1, CA2: *Candida albicans* 2, CA3: *Candida albicans* 3, CA4: *Candida albicans* 4, MIC: minimum inhibitory concentration, and “—” no activity.

## Data Availability

All data are included in the manuscript.
